# A case study of the counterpart technical support policy to improve rural health services in Beijing

**DOI:** 10.1186/1472-6963-12-482

**Published:** 2012-12-29

**Authors:** Weiyan Jian, Kit Yee Chan, Shunv Tang, Daniel D Reidpath

**Affiliations:** 1 Department of Health Policy and Management, School of Public Health, Peking University Health Science Centre, 38 Xueyuan Road, Haidian District, Beijing, 100191, China; 2 Nossal Institute for Global Health, University of Melbourne, Carlton, Victoria, 3010, Australia; 3 School of Medicine and Heath Sciences, Jalan Lagoon Selatan, Bandar Sunway, Selangor, DE, 46150, Malaysia

**Keywords:** Rural–urban health service inequality, Counterpart technical support policy, Health service reform, Health services accessibility, Healthcare disparities, Rural population, Urban population, National health programs, China

## Abstract

**Background:**

There is, globally, an often observed inequality in the health services available in urban and rural areas. One strategy to overcome the inequality is to require urban doctors to spend time in rural hospitals. This approach was adopted by the Beijing Municipality (population of 20.19 million) to improve rural health services, but the approach has never been systematically evaluated.

**Methods:**

Drawing upon 1.6 million cases from 24 participating hospitals in Beijing (13 urban and 11 rural hospitals) from before and after the implementation of the policy, changes in the rural–urban hospital performance gap were examined. Hospital performance was assessed using changes in six indices over-time: Diagnosis Related Groups quantity, case-mix index (CMI), cost expenditure index (CEI), time expenditure index (TEI), and mortality rates of low- and high-risk diseases.

**Results:**

Significant reductions in rural–urban gaps were observed in DRGs quantity and mortality rates for both high- and low-risk diseases. These results signify improvements of rural hospitals in terms of medical safety, and capacity to treat emergency cases and more diverse illnesses. No changes in the rural–urban gap in CMI were observed. Post-implementation, cost and time efficiencies worsened for the rural hospitals but improved for urban hospitals, leading to a widening rural–urban gap in hospital efficiency.

**Conclusions:**

The strategy for reducing urban–rural gaps in health services adopted, by the Beijing Municipality shows some promise. Gains were not consistent, however, across all performance indicators, and further improvements will need to be tried and evaluated.

## Background

The world has experienced an unprecedented level of urbanization over the past 50 years, with the world’s urban population exceeding the rural population for the first time in the early years of this century. For health ministries, urbanization has the advantage of geographically concentrating the demand for services, and providing opportunities for more cost effective and more accessible health care provision. The corollary of this is a likely inequality arising between the health service provision available in urban and rural areas, with rural areas experiencing poorer, less accessible services [e.g. [1-6]. The issue has particular significance for China because of the absolute size of its population, the rapid economic growth it has enjoyed over recent years (with the characteristic increase in urbanization), and the fact that 50% of the population continues to live in rural areas [7].

In response to the concern about the rural–urban inequality, the government of China has, since 1997, focused health systems reform on narrowing this gap [8]. Of the 21.7 billion Chinese Yuan (~3.4 billion USD) China had planned to invest into its health services development between 2006 and 2010, approximately 68% was marked for building capacity in rural health [9]. Counterpart technical support policies aimed at mobilizing the greater capacity of top urban hospitals to improve rural hospital capacity have been in development since 2005. It commenced when the Ministry of Health, Ministry and Finance and the Bureau of Traditional Chinese Medicine jointly established the “Mobilization of 10,000 Doctors for Rural Health Project” in Western and Central China, and began as a pilot in 2009 [10]. Services in rural communities have also been integrated into urban hospital policies which require urban doctors to serve in rural hospitals for an accumulated period of 12 months before being eligible for promotion [11]. More recent policy developments have focused on the establishment of longer-term partnerships between rural and urban hospitals to strengthen rural hospital capacity [12].

Progress for implementing the national agenda for reducing the rural–urban gap in health services varies across China’s many administrative units. Beijing, the nation’s capital, has been at the forefront of the country’s rural health services reform, and serves as an exemplar case study for health reform in other parts of China. With an area of 16,801.25 km^2^ (the size of Wales) and a population of 20.19 million as of 2011, Beijing municipality is divided into five urban districts, eight suburban districts, and two rural counties [13]. Urban Beijing is serviced by 13 general hospitals, while the 10 suburban and rural areas are serviced by 11 county hospitals. Implementation of rural support initiatives in Beijing can be traced back to 2003 before the counterpart technical support policy was formalized on a national level [14].

Beijing’s commitment to the counterpart initiative has gradually increased [15]. In 2008, the Beijing Municipal Health Bureau formalized the partnership between all 24 hospitals targeted by the counterpart technical support policy. Memoranda of understanding (MOU) were signed between the 11 recipient rural hospitals and their 13 supporting urban partners. Nine recipient rural hospitals signed the MOU with nine urban supporting hospitals, while the remaining two rural hospitals formed partnerships with two urban hospitals each. The MOU covered three elements: (1) physicians from the supporting urban hospitals must each serve at least one month of every year in the recipient rural hospitals; (2) supporting hospitals must provide appropriate training to staff members of their recipient hospitals in areas where the recipient hospitals lack clinical capacity; (3) when a recipient hospital encounters clinical difficulties, external consultation must be provided by its partner urban hospital, and if necessary, have the patient transferred to the partner hospital.

An annual budget of approximately 2,500,000 yuan was set aside by the Beijing Municipal Health Bureau to remunerate the urban hospitals for their participation [16]. The precise amount payable each year was dependent on the feedback contained in the recipient hospitals’ annual reports. By the end of 2010, approximately 1,200 doctors from the participating urban hospitals were mobilized to provide 50,000 workdays to their rural counterparts [16]. During this period, the Chinese government’s general funding for rural hospitals was increased by 50.8%, while funding for large medical equipment purchase in rural hospitals was increased by 40.7%. This investment was in sharp contrast to funding provided for urban hospitals in the same period which was marked by a 24.4% increase in general funding, and 1.7% increase in funding for equipment purchases [17,18].

This paper aims to evaluate the latest development of Beijing’s counterpart technical support policy from early 2008, when Beijing’s 13 leading urban general hospitals were appointed to each form partnerships with one appointed leading rural general hospital [19]. Using hospital records from 2008 to 2010, this paper focuses on understanding changes in rural hospital capacity and services during the period of reform. More specifically, we compare the 2008 data from participating rural hospitals with the 2010 data. The following areas were compared: the scope of hospital services, medical safety, treatment success of difficult cases, length of inpatient stay, and treatment costs. For comparison purposes, changes in hospital capacity and services for the urban counterpart hospitals were also analyzed, specifically looking at the changes in the rural–urban gap in health services.

## Methods

### Source of data

This study draws upon data from the “Beijing Database of Discharged Patients’ Basic Medical Case Records” managed by the Beijing Public Health Information Centre. The database records information on case diagnoses, procedures, admission time, medical costs and cost structure, and the patient's general individual characteristics (e.g. age, sex, birth weight). Data for the years 2008 and 2010 were extracted for the 24 (11 urban, 11 rural) participating hospitals, totaling 1,639,133 cases.

### Risk-adjustment tools and indicators

As the participating hospitals differ in their disease treatment profiles, “risk adjustment” is necessary to establish the comparability of cases between hospitals. In this study, risk adjustments were conducted using Diagnosis Related Groups Beijing Version (BJ-DRGs) [20-22]. Six indicators were established:

1. DRG volume, which reflects the scope of conditions treated by different hospitals. The larger the DRG volume, the larger the scope of conditions treated.

2. Case-mix index (CMI), a standard measure used for comparing the severity of patients’ illnesses between hospitals. A high CMI score indicates that a given hospital has treated many patients with severe illnesses [23,24].

3. Cost Efficiency Index (CEI), which allows comparisons of the costs for treating the same disease categories between different hospitals. High CEI scores indicate high cost of treatment and low cost efficiency.

4. Time Efficiency Index (TEI), which allows comparisons of the time required for treating the same disease categories between different hospitals. High TEI scores indicate lengthy treatment time and low time efficiency.

5. Mortality rates of low risks cases – this refers to the mortality rate of each hospital for cases with conditions that have a low probability of death. The indicator is used to reflect hospital safety.

6. Mortality rates of high-risk cases – this refers to the mortality rate of each hospital for cases with conditions that have a high probability of death. The indicator is used to reflect hospital capability for treating emergency cases and severe cases.

The analysis follows a similar method to our earlier studies using similar hospital data sources [22,25,26]. The following formula was used to calculate the case mix index, in which, h is the hospital for which the index was being calculated; *W*_*g*_ is the weight associated with the *DRG*_*g*_ (as set out by the BJ-DRGs system [20]; *n*_*gh*_ is the number of cases in the *DRG*_*g*_ in hospital h; and *n*_*gn*_ is the number of cases in the *DRG*_*g*_ of the entire sample [27]:

(1) $$\text{CMI}=\frac{\left[\sum_{g}W_{g}\times n_{\text{gh}}\right]/\sum_{g}n_{\text{gh}}}{\left[\sum_{g}W_{g}\times n_{\text{gn}}\right]/\sum_{g}n_{\text{gn}}}$$

The following formula was used to calculate the cost efficiency index, in which, *n*_*j*_ is the number of cases in *DRG*_*j*_ in the hospital; *k*^*c*^ (or *k*^*d*^) means charge per case (or average length of stay) within each *DRG*_*n*_ that hospital dividing charge per case (or average length of stay) within each DRG based on full sample size:

(2) $$\text{CEI}=\frac{\sum_{j}k_{j}^{c}n_{j}}{\sum_{j}n_{j}},\text{TEI}=\frac{\sum_{j}k_{j}^{d}n_{j}}{\sum_{j}n_{j}}$$

We computed the inpatient mortality of each DRG (*M*_*i*_), took the logarithm of *M*_*i*_ (*Ln(M*_*i*_*)*) and calculated the mean $\overset{}{\text{Ln}\left(M_{i}\right)}$ and standard deviation (*s*_*i*_) of *Ln(M*_*i*_*)*. “Low-risk-cases” were operationalized as DRGs with *Ln(M*_*i*_*)* lower than $\overset{}{\text{Ln}\left(M_{i}\right)}$ minus 1*s*_*i*_, while “high-risk cases” were operationalized as those with higher than $\overset{}{\text{Ln}\left(M_{i}\right)}$ plus 1*s*_*i*_.

### Models for assessing the change in rural–urban gaps

The statistical significance of the change in the rural–urban utilization gap was estimated separately for each of the six indicators (*Y*) in turn. The regression model was:

(3) $$Y=\beta_{0}+\beta_{1}\text{Time}+\beta_{2}\text{Rural}+\beta_{3}\text{Time}*\;\text{Rural}{+}_{Ε}$$

in which, *Time* is a dummy variable (*Time* = 1 if the year is 2010, and 0 otherwise); *Rural* is a dummy variable (*Rural* =1 if the patient is from a rural hospital, and 0 otherwise); the interaction effect Time*Rural represents the change in the rural–urban utilization gap. This approach is replicated, *mutatis mutandis*, for the six indicators.

### Ethics

The approach taken to accessing and utilizing the hospital data was analogous to the National Health Service in England's system for allowing researchers to hospital episodes statistics (HESonline). The data was released following a formal request to the Beijing Public Health Information Centre which assessed the risk and sensitivity of the request following normal internal procedures. A waiver was granted by the Human Ethics Committee of Peking University Health Sciences Center for this study (Waiver ID: 2012009).

## Results and discussion

As a preliminary approach, graphical and descriptive techniques were used to establish the background.

### Descriptive results

#### a. Changes in the scopes of disease types and technical skills requirements

**Figure 1:**
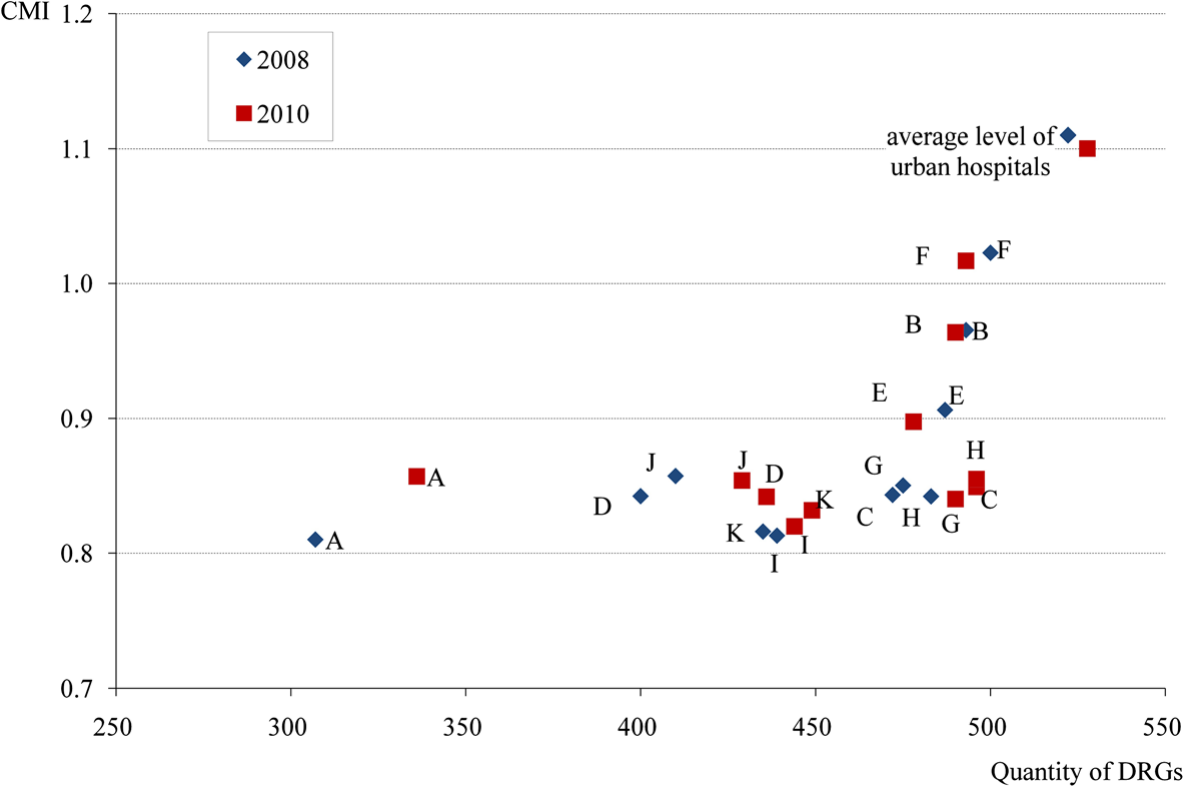
Changes in DRG and CMI of the 11 rural Beijing hospitals between 2008 and 2010.

Figure 1 shows a scatter plot of CMI by DRG volume for the 11 rural hospitals in 2008 (blue diamond’s) and 2010 (red squares). For reference purposes the average of the 13 urban hospitals is also shown on the plot. The 11 rural hospitals showed large variation in the scope of conditions (as measured by DRG volume) and the severity of the cases they treated (as measured by CMI) at baseline. Hospital A in 2008, for instance, had a DRG volume of 307 and CMI of 0.81, while Hospital F had a DRG volume of 500 and a CMI of 1.02. There was an average increase of 12 DRGs in the scope of conditions (as measured by DRG volume) for rural hospitals between 2008 and 2010. Hospital D had the largest expansion in case scope (an increase of 36 DRGs during this period), followed by hospitals A (29 DRGs) and C (24 DRGs). However, sizable changes in the average case severity (as measured by CMI) were observed only for hospital A which also happened to be the hospital with the lowest DRGs both just before and 2 years after the introduction of the policy. No obvious changes in CMI were observed in the other 10 participating rural hospitals. The least improvement in CMI and DRG was observed in hospitals B and F, which already had the highest CMI and DRG scores before the introduction of the policy. Despite these improvements, all 11 rural hospitals measured worse than the urban hospital average, and this was true both at baseline and two years after the introduction of the policy.

#### b. Changes in medical expenses and inpatient time for the 11 rural Beijing hospitals

**Figure 2:**
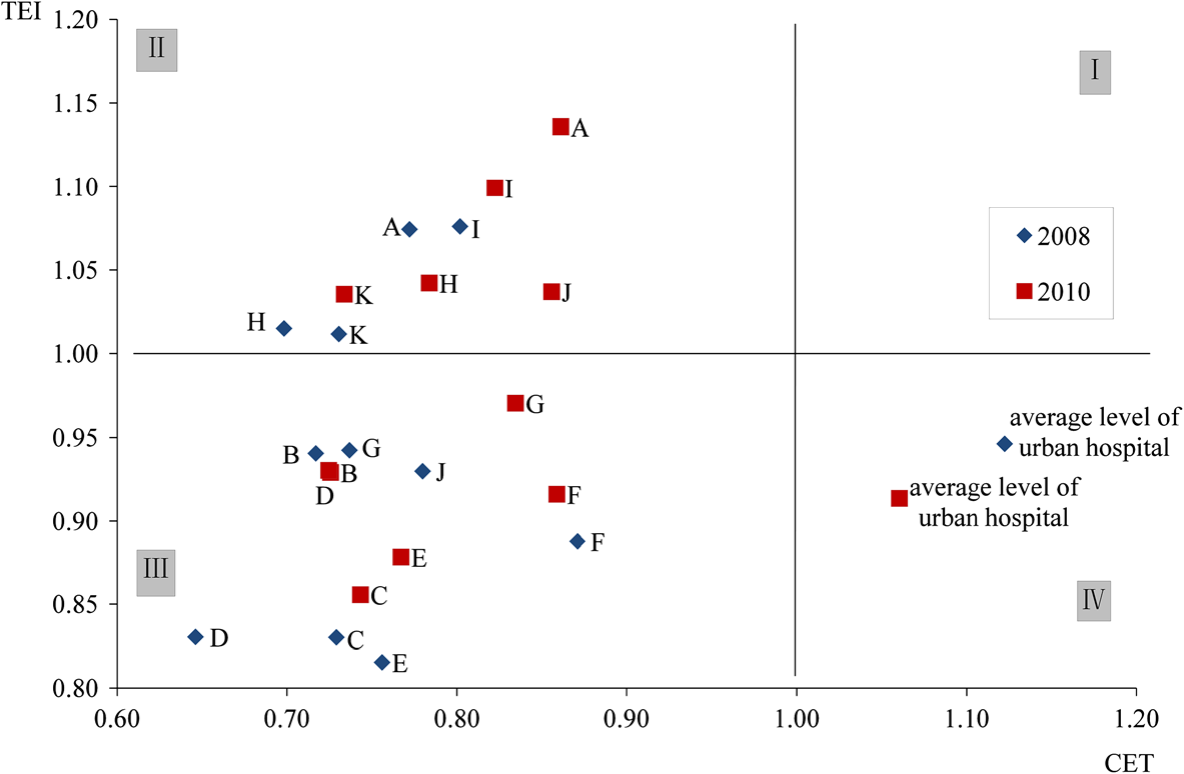
Time efficiency (TEI) and cost efficiency (CEI) changes for patients treated in rural hospitals 2008–2010.

Figure 2 shows a scatter plot of cost (CEI) and time (TEI) efficiencies for the 11 rural hospitals in 2008 (blue diamond’s) and 2010 (red squares). For reference purposes the average of the 11 urban hospitals is also shown on the plot. The graph is divided into quarters by a vertical line at CEI=1 and a horizontal line at TEI=1. The first quarter (I) in the upper right-hand quadrant is indicative of low time and cost efficiency (i.e., higher costs and longer stays); the second quarter (II) in the upper left-hand quadrant is indicative of low time but high cost efficiency (i.e., longer stays, but lower costs); the third quarter (III) in the lower left-hand quadrant is indicative of high time and high cost efficiency (i.e., shorter stays and lower costs); the fourth quarter (IV) in the lower right-hand quadrant is indicative of high time, but low cost efficiency (i.e., shorter stays, but higher costs).

All rural hospitals fell into quadrants II and III throughout the period 2008–2010, indicating relatively low cost expenditure but the use of varying lengths of hospital stay between hospitals for treating the same disease type. The urban hospital averages for the same period fell into quadrant IV, indicating the higher cost of treatment of urban hospitals relative to rural hospital, and the tendency for urban hospitals to keep inpatients for shorter lengths of time.

Nine of the 11 rural hospitals had reduced time efficiency following the implementation of the policy. In 2008, the average CEI and TEI for the 11 rural hospitals were 0.75 and 0.94 respectively, two years post-policy, the cost and time for treatment were increased to 0.79 and 0.98 respectively, indicating an increase in both the cost of treatment and the length of stay. The most significant efficiency reductions were observed in hospitals A, D, G, H and J. Hospital J for instance went from a TEI of 0.93 and CEI of 0.78 in 2008 (quadrant III) to a TEI of 1.04 and CEI of 0.86 in 2010 (quadrant II). Hospital K had a small increase in hospital stay without altering cost, while hospital F had a small increase in hospital stay at a slightly lower cost. In contrast, on average urban hospitals had improved both in terms of cost and time efficiency during this period, although they remained less cost efficient than the rural hospitals. In 2008, the average CEI and TEI of the 13 urban hospitals were 1.12 and 0.95 respectively, compared with the average CEI and TEI of 1.06 and 0.91 in 2010.

#### c. Changes in rural hospital safety records

**Figure 3:**
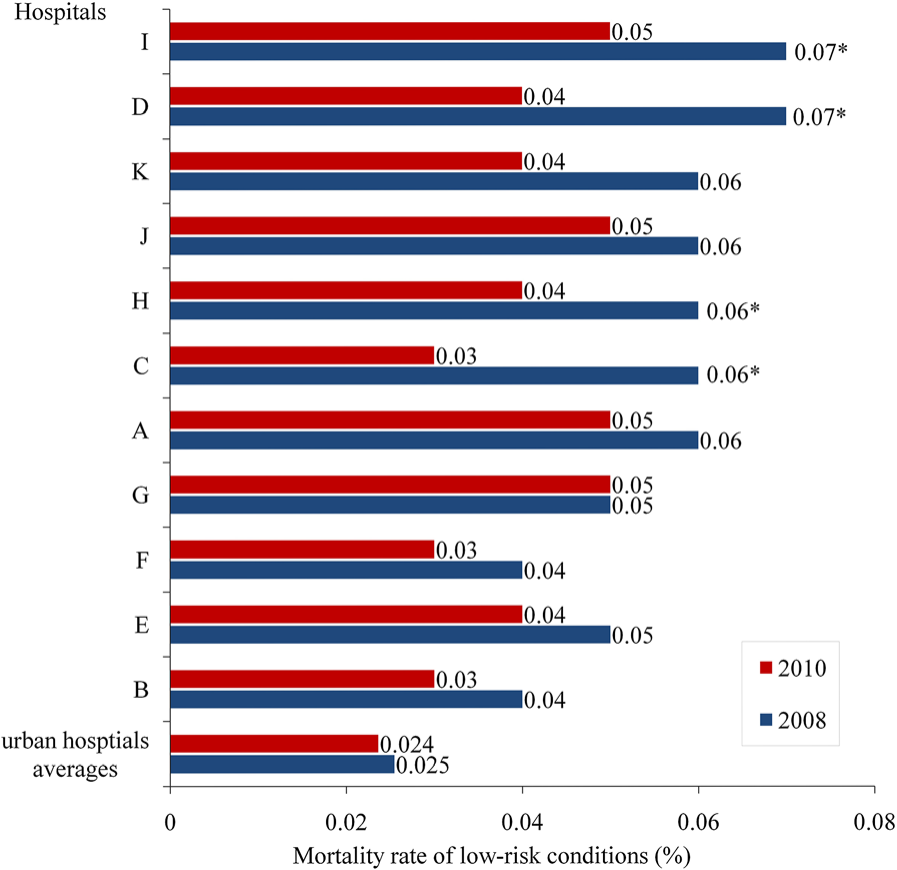
Changes in low-risks disease mortality rates in Beijing’s rural hospitals (2008–2010).

Figure 3 is a bar chart showing changes in mortality rates for low-risks-conditions between 2008 (blue bars) and 2010 (red bars) in each of the 11 rural hospitals, and for the average urban hospital. The immediately noticeable difference between hospitals is the much lower mortality rate in the average urban hospital than in any of the rural hospitals. The second point to observe is that with the exception of hospital G, every hospital (including the urban average hospital) showed reductions in the mortality rate between 2008 and 2010.

#### d. Changes in hospital capacity for treating emergency and severe cases

**Figure 4:**
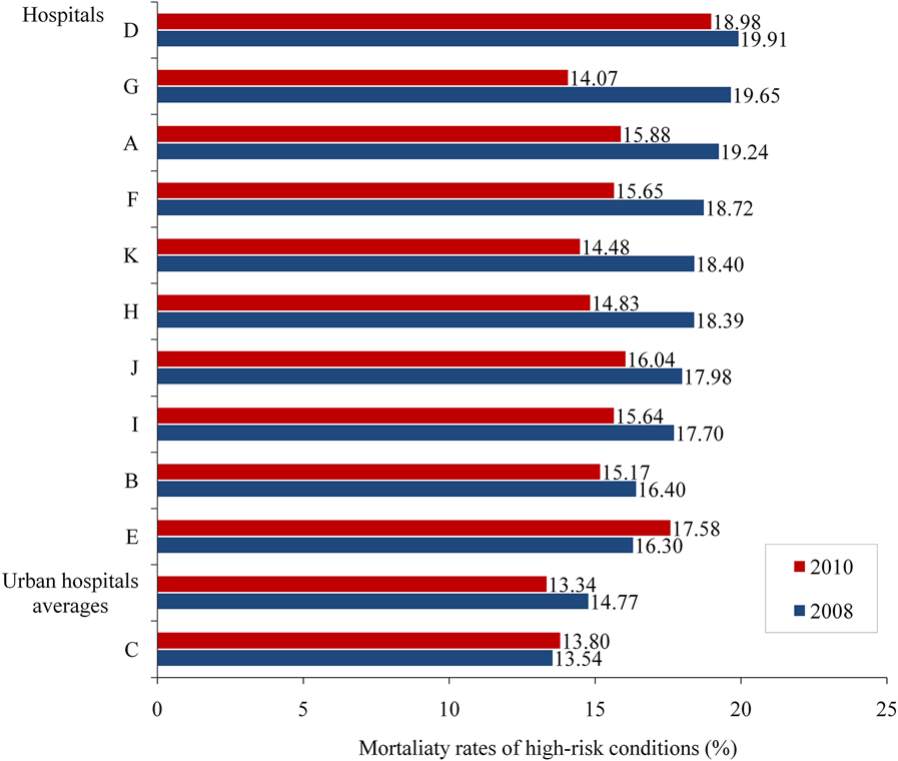
Changes in the morality rate for high-risks-cases in Beijing rural hospitals (2008–2010).

Figure 4 shows an equivalent mortality analysis for high-risk cases. With the exception of Hospital E and the average urban hospital, there were reductions in the mortality rates associated with high-risk cases, with the largest reductions, in descending order, for hospitals G, K, H, A, and F.

### Regression analyses for rural–urban gaps in health services capacities

Results of the regression analyses for the health services capacity as measured by the six indicators are shown in Table 1. Using the 13 urban hospitals as benchmark, between 2008 and 2010, significant reduction in rural–urban gaps were observed both in terms of diversity of cases treated as measured by DRG volume (−2.27, p<.001) and in treating severe and acute cases as measured by MHR (−0.11, p<.001).While there was a statistically significant reduction in the rural–urban gap in terms of medical safety, this reduction was small (MLR=−0.04, p<.001). In contrast, there were no significant changes in rural–urban gaps observed in terms of the complexity of cases treated (CMI) or time efficiency (TEI) during this period. The costs for treating the same disease categories between hospitals (CEI) had reduced considerably for urban hospitals, but has risen significantly for rural hospitals (0.11, p<.001).

**Table 1:** Regression results of changes in rural–urban health services capacity (2008–2010)

	**Range of cases treated (DRGs volume)**	**Complexity of cases treated (CMI)**	**Cost efficiency (CEI)**	**Time efficiency (TEI)**	**Medical safety (MLR) (poisson)**	**Capacity in treating severe and acute cases (MHR) (logistic)**
Changes in rural–urban gaps	−2.27*	0.02	0.11*	0.08	−0.04*	−0.11*
	(6.27)	(0.12)	(0.04)	(0.06)	(0.03)	(0.05)
Time change	15.55*	−0.01	−0.06*	−0.03	−0.07	−0.04*
	(10.57)	(0.09)	(0.03)	(0.04)	(0.04)	(0.98)
Rural–urban gaps	−66.64**	−0.23*	−0.37 **	−0.01	0.13**	0.29**
	(18.57)	(0.09)	(0.03)	(0.04)	(0.06)	(0.98)
Prob. > F	0.000	0.000	0.000	0.000	0.000	0.000
Adj. R^2^	0.456	0.388	0.896	0.255	——	——

### Reasons for the varying performance between the four indicators

In the three years between 2008 and 2010, two policies were targeted at rural health organizations: (1) financial and machinery investment; and (2) “counterpart technical support”. The former focused on financial resources, while the latter focused on technical resources. From the perspective of the inputs and the outputs, it is reasonable to believe the counterpart technical support policy was at least in part responsible for closing the gap in technical expertise between rural and urban hospitals during this period. Assuming this is correct, overall, our current analysis shows that, in the two-years following the introduction of the counterpart technical support policy, there have been promising improvements in rural hospital services, particularly in terms of the expansion in the scope of services provided by participating rural hospitals and their capacity to treat emergency cases. Medical safety records of some of the participating rural hospitals have also improved following the introduction of the policy. However, large rural–urban gaps still remain in all of these areas. Furthermore, the counterpart support policy appeared to be unsuccessful in improving the capacity of rural hospitals to treat severe illnesses. The current analysis showed that the cost and time efficiencies of the participating rural hospitals had decreased in the two years following the introduction of the policy, but this was offset by greater improvements observed in urban hospitals, further widening the rural–urban gap.

These unexpected and conflicting trends observed between rural and urban hospitals in time and cost efficiencies could be associated with three inter-related factors. Firstly, the reduction in cost and time efficiency in rural hospitals could be directly related to the heavy government investment in machinery purchases for rural hospitals (e.g. [28]). While the availability of new equipment has allowed more accurate diagnoses and sophisticated treatment for rural patients, it could also have led to more tests and services that were previously not available, leading to lengthier treatment time and increased treatment cost [29,30]. Secondly, the counterpart technical support received by rural hospitals also brought new skills from urban hospitals. These new skills are often accompanied by expensive medical consumables (e.g. stents) [31]. These skills while essential to bridging the gap in treatment received by rural and urban patients, may also contribute to increasing the cost of treatment for rural patients. Thirdly, improvements in time and cost efficiencies for urban hospitals could have been due to successes from their recent internal managerial reforms which emphasized the need to reduce the length of hospital stay for inpatients and overall hospital efficiency through improvements in hospital management [32,33]. Thus far, the counterpart technical support policy has focused only on the transfer of medical skills from urban to rural doctors while overlooking the need to transfer management expertise [34].

Investment in new technology and machines in rural hospitals has been an important part of China’s strategies for closing rural–urban gaps in health services [35]. Our results indicate, however, that if the process is not managed properly, the approach could substantially increase the cost of health care in the future. In addition, the transfer of medical skill and expensive hardware without the transfer of the model of management and managerial skill could be counterproductive to service efficiency. Improving rural hospital management might be the next essential step that will allow the counterpart support program to achieve better use of medical resources for rural hospitals.

### Counterpart technical support–a solution for all rural areas?

The current analysis shows that not all rural hospitals benefited equally from the counterpart technical support policy. This was especially true for rural hospitals that started from a considerably lower capacity baseline before the reform. Hospital A is a case in point, where 2 years after the introduction of the counterpart support policy, the hospital was still treating less than 350 DRGs, which indicates that the residents serviced by this hospital were not receiving sufficient treatment. In the on-going discussion of the future direction of China’s health system reform, a recent suggestion for reducing the rural–urban service gap was the establishment of rural branches of leading urban hospitals [36]. We understand that this solution is currently being implemented elsewhere in China (e.g. Ningxia), and its successes is subject to evaluation [37]. If implemented properly, we believe this could be a solution for improving rural health services in areas that are severely lagging behind. However, the decision to establish new rural branches of urban hospitals or to adopt the counterpart technical support policy should be based on a thorough assessment of existing rural hospital capacity. Furthermore, new rural hospital branches, should not to make existing rural hospital redundant. A collaborative and consultative approach is therefore needed with the existing rural hospital before a new urban hospital branch is established in the same location. The assessment should thus identify areas of strengths and weaknesses of the existing rural hospital, so that the new hospital can focus on offering services that support rather than compete with existing services.

In the attempt to improve rural health services, governments in many countries have experimented with different strategies to retain medical workers in rural areas. This has included the use of financial incentives and support infrastructure improvements for rural health workers, modifications to medical education curriculums to improve understanding of rural health, and medical school admission targeted at students from rural areas [38]. Many of these strategies are long-term strategies, and their effect can only be reviewed over extended periods of time. A limitation of the current study is the relatively short duration between policy implementation and evaluation.

Furthermore, it is likely that different combinations of strategies work differently under different circumstances. The policy of retaining medical workers in rural areas through improved financial incentives might be straightforward in some contexts; but in China, it would involve complex collaborations between multiple ministries and government departments that control different aspects of the income of hospitals and medical doctors. The strategy has been discussed in the course of China’s on-going health system reform, but is difficult to implement without substantial reform to the overall governmental structure itself. The current study shows that the counterpart technical support policy might offer a promising way for improving rural hospitals service capacity within the Chinese context within a relatively short timeframe. Equally importantly, the collaborative relationships between the participating rural and urban hospitals generated by this policy are long-term. If implemented properly, the policy could continue to yield positive changes in the years to come.

Future policy development needs high quality research that monitors the impact of policy changes overtime (e.g. [39]). The current analysis suggests that in order to maximize the policy impact, a more holistic approach should be taken in the future, where the transfer of models of management and managerial skill is included, in addition to the transfer of medical skills and technology. In the short-term, the policy should focus on transferring skills crucial to the three areas of weakness identified in this study: medical safety, treatment of difficult cases and improving time efficiency.

### Limitations

A limitation of the current study was that while the data shows the variation in the changes in hospital capacities according to the specific indicators, it does not identify the reasons for the variation. This merits further research using qualitative methods. It was difficult to determine from the data how much of the improvement that was identified was attributable to real advances in the clinical capacity of rural doctors, and how much of it was due to the participation of urban doctors. The focus of the study was on inpatient service capacity. Outpatient services contribute to approximately one-third of all hospital services and fall within the scope of the counterpart technical support policy. A full assessment of the policy therefore should also consider outpatient services.

The Chinese government’s enthusiasm and investment into the country’s health reform has not been complimented by well-designed evaluation that inform questions of effectiveness and its likely impact in other parts of China. Most evaluations in the literature thus far have been based on opinions (commentary) instead of data. In the absence of well-designed evaluations, it is difficult to provide insights into the effectiveness and real progress of the reform for informing future policy development.

## Conclusions

Imbalances in health service capacity between rural and urban areas is a key reason for health service inequity in many countries. For a rapidly developing country such as China which has the world’s largest population and a substantial landmass, socio-economic imbalances including health services inequity are readily apparent [40]. Such imbalances in development have the potential for limiting the country’s future socio-economic progress [41]. Human resource, financial and material investments into the country’s economically less developed areas is an important means of addressing inequities in medical care. To this end, the Chinese government’s recent investments to address the rural and urban health services gaps have been substantial [9]. Using the nation’s capital as a case study, we evaluated the results of one such government initiative – the counterpart technical support policy. We have shown that the policy might have positively contributed to improving the skills of medical workers, the case-mix and survival rates in most rural contexts. However, further improvement is required in cost and treatment efficiency. The transfer of models of management and managerial skill could be a key to such improvements, while the current government focus on investing expensive medical equipment may simply push up the cost of health care for rural areas. The policy may not be the most efficient way of improving rural health services in areas where existing services capacity is too low.

## Abbreviations

CMI: Case-mix index; CEI: Cost expenditure index; DRG: Diagnosis Related Groups; TEI: Time expenditure index.

## Competing interests

The authors declare that they have no competing interests.

## Authors’ contributions

JWY conceptualized the paper, negotiated data access, oversaw the study design, data analysis and the drafting of the paper. KYC contributed to the data analysis and interpretation, and led the writing of the paper. ST contributed to the literature review and data analysis, and participated in the drafting of the paper. DDR contributed to the data analysis, data interpretation and writing of the paper. All authors read and approved the final manuscript.

## Pre-publication history

The pre-publication history for this paper can be accessed here:

http://www.biomedcentral.com/1472-6963/12/482/prepub
